# Inverted 'V' osteotomy excision arthroplasty for bony ankylosed elbows

**DOI:** 10.1186/1749-799X-6-60

**Published:** 2011-12-05

**Authors:** Chadrabose Rex, Rameshkumar Periyasamy, Subbachandra Balaji, Premanand C, Shreyas Alva, Shiva Reddy

**Affiliations:** 1 Department of orthopaedics, Rex Ortho Hospital, Number 43, RR Layout, Coimbatore 641045, India

## Abstract

**Background:**

Bony ankylosis of elbow is challenging and difficult problem to treat. The options are excision arthroplasty and total elbow replacement. We report our midterm results on nine patients, who underwent inverted 'V' osteotomy excision arthroplasty in our hospital with good functional results.

**Materials:**

Our case series includes 9 patients (seven males and two females) with the mean age of 34 years (13-56 years). Five patients had trauma, two had pyogenic arthritis, one had tuberculous arthritis, and one had pyogenic arthritis following surgical fixation.

**Results:**

The average duration of follow up is 65 months (45 months-80 months). The mean Mayo's elbow performance score (MEPS) preoperatively was 48 (35-70). The MEPS at final follow up was 80 (60-95). With no movement at elbow and fixed in various degrees of either flexion or extension preoperatively, the mean preoperative position of elbow was 64°(30°to 100°). The mean post operative range of motion at final follow up was 27°of extension (20-50^0^), 116°of flexion (110^0^-130^0^), and the arc of motion was 88°(80^0^-100^0^). One patient had ulnar nerve neuropraxia and another patient developed median nerve neuropraxia, and both recovered completely in six weeks. No patient had symptomatic instability of the elbow. All patients were asymptomatic except one patient, who had pain mainly on heavy activities.

**Conclusion:**

We conclude that inverted 'V' osteotomy excision arthroplasty is a viable option in the treatment of bony ankylosis of the elbow in young patients.

## Introduction

Bony ankylosis of elbow is not uncommon. The conditions causing bony ankylosis are trauma, head injury, inflammatory arthritis, infection, burns, and neurological conditions, like hemiplegia, anterior poliomyelitis, and idiopathic [1-5]. The challenge lies in treating such patients as the options are limited and are associated with complications. The total elbow arthroplasty (TEA) is increasingly done for various conditions of the elbow including bony ankylosis[6,7]. TEA in elbow is associated with high complication rate, which varies from 26% in ankylosed elbows[6] to as high as 44%[8], in elbows with various etiologies. Though the complications rates are decreasing in TEA, the consequences secondary to complications are far reaching and are difficult to address[9]. Thus neither the cost of the implants, and nor the high complications associated with TEA has made it popular in developing countries. Many authors have used excision arthroplasty [10,11] to regain functional motion in ankylosed and stiff elbows. We used a modified excision arthroplasty, where we resected the bone in inverted v shape, to treat our patients. The objective of our case series is to analyze the functional outcome and the complications associated with our modified excision arthroplasty for bony ankylosis of the elbow.

### Patients and methods

From 2000-2005, 47 patients with elbow ankylosis were treated in our hospital. Nine patients had bony ankylosis, and thirty eight, fibrous ankylosis. The patients with bony ankylosis were included in the study. None of these patients had active soft tissue or bone infection at the time of the procedure. Five patients had moderate pain on activity over shoulder girdle, two patients had mild pain, and two patients were asymptomatic. The primary indication for the procedure is functional restriction of the patients in seven and both functional limitation and pain in two patients. The cause of the pain in two patients may be assumed to occur due to the compensatory movements at adjacent joints or due to post infective and the exact cause is difficult to be elucidated as it involves the whole of upper extremity inconsistent in location and duration. Thus, all nine patients with bony ankylosis were treated with inverted "V" osteotomy excision arthroplasty. Seven were males, and two females. The mean age of the patient was 34 years (13-56 years). The right hand was involved in four patients and the left hand in five patients. All were right handed dominant patients. The mean duration of ankylosis before excision arthroplasty was 7 years (2-15 years). Six patients had at least one previous surgery, with two patients having had three surgeries prior to the index procedure (table 1).

**Table 1:** clinical data of nine patients with bony ankylosis and arc of motion at final follow up

No	sex	age	pre op diagnosis	side	pre surgical treatment	duration of stiffness (Years)	duration of follow up(months)	Preoperative position of arm	ROM at final follow up	arc of motion at final follow up
1	F	28	post infective	R	I&D #	15	80	80	50-130	80

2	F	40	Tuberculosis	R	open biopsy	9	68	40	30-110	80

3	M	13	post infective	R	nil	2	70	60	30-110	80

4	M	26	trauma	L	native treatment $	8	78	30	20-120	100

5	M	56	trauma(swide swipe injury)	R	repeated surgeries	3	72	100	40-120	80

6	M	32	machine inj	L	repeated surgeries	4	67	80	40-130	90

7	M	37	trauma	L	surgically fused	11	55	90	30-130	100

8	M	44	Trauma with post operative infection	L	plating	2	52	70	20-110	90

9	M	34	trauma	L	native treatment $	12	45	30	20-110	90

#- incision and drainage

$- native treatment includes treatment by unqualified personnel and varies from oil massage to splinting using wooden sticks

### Operative Technique

**Figure 1:**
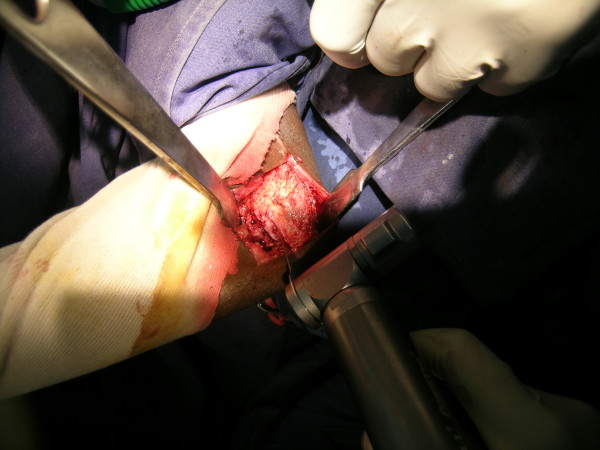
**Intraoperative photograph demonstrating lateral limb of osteotomy being performed through lateral approach**.

**Figure 2:**
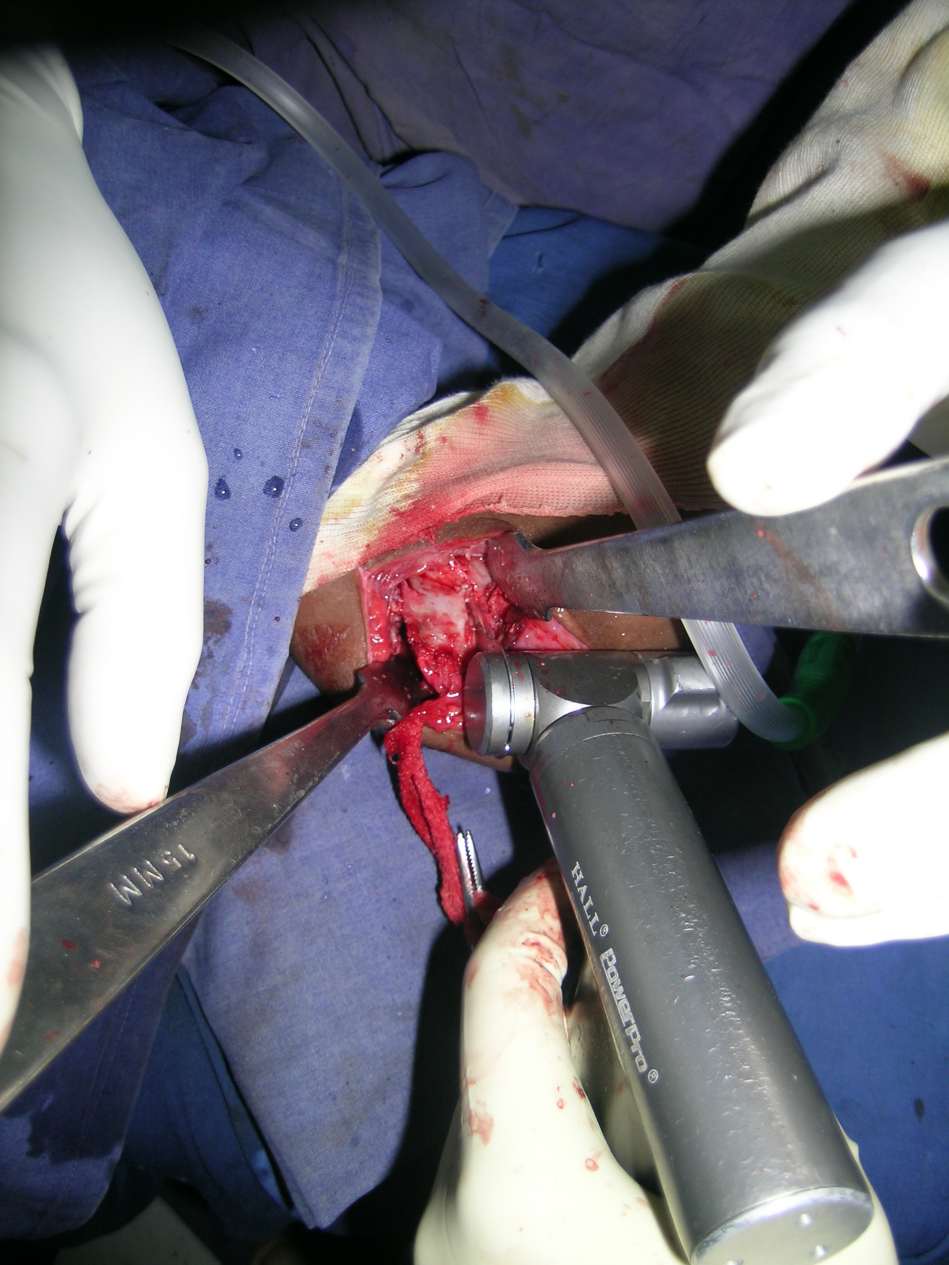
**Intraoperative photograph demonstrating medial limb of osteotomy being performed through medial approach**.

**Figure 3:**
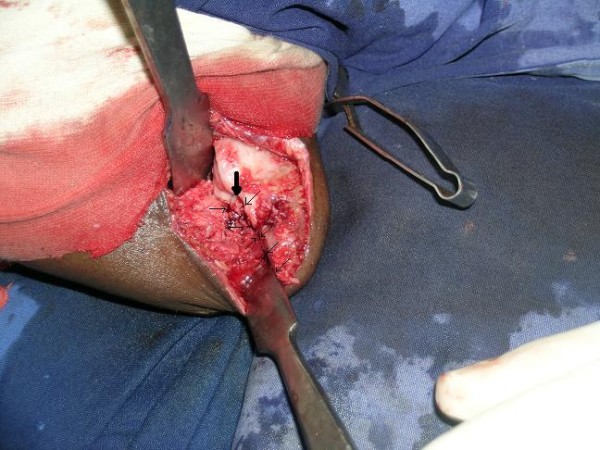
**V shaped osteotomy seen from lateral side with thin arrows showing the outline of V osteotomy and thick arrow showing the apex of the osteotomy in the humerus**.

**Figure 4:**
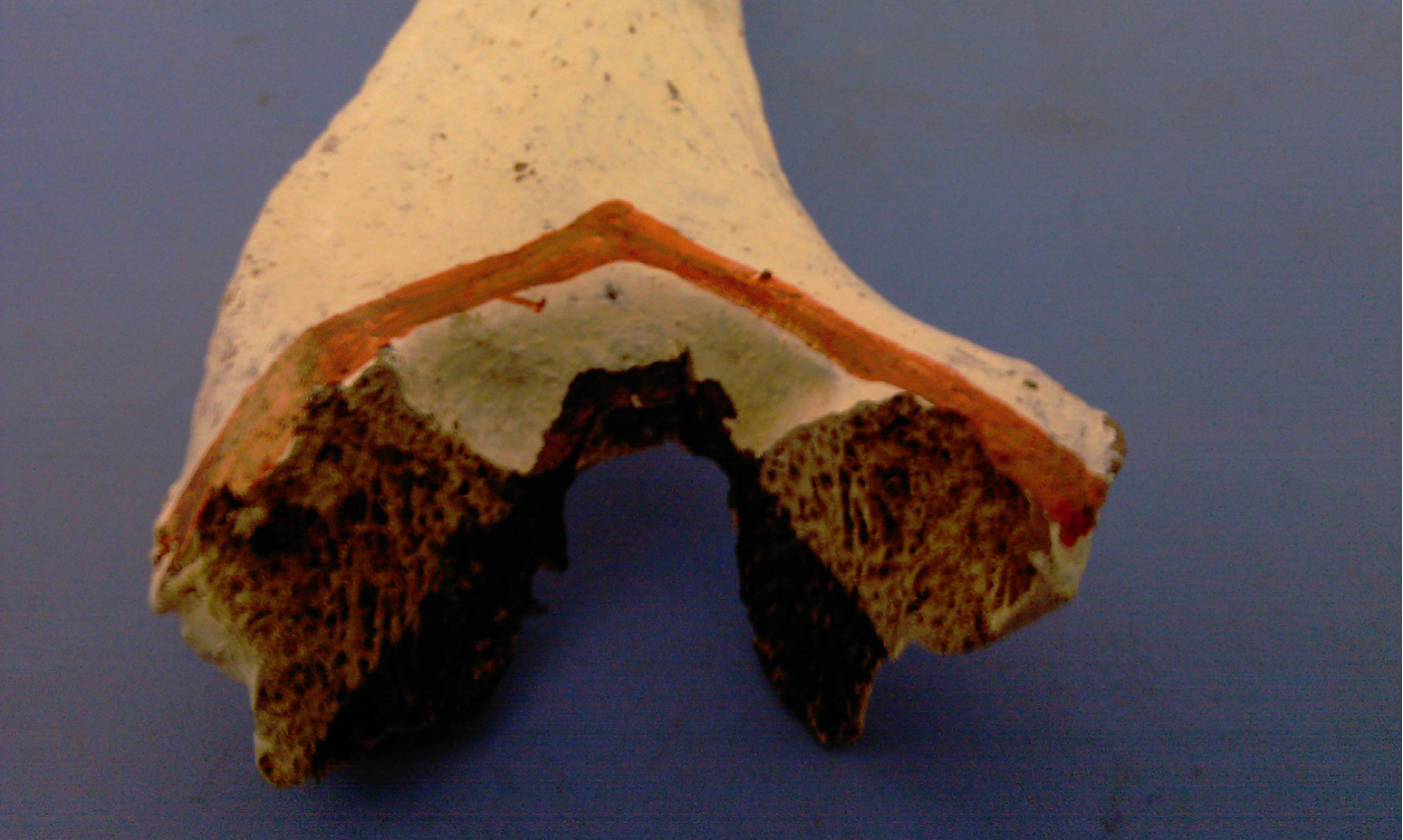
**Bone model showing the v osteotomy**.

**Figure 5:**
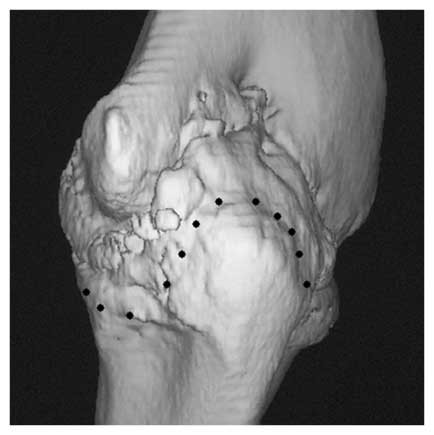
**Line diagram showing the osteotomy lines**.

Patients were administered regional anesthesia (scalene block), and put in supine position with tourniquet control. All patients were operated by combined medial and lateral approach with two separate mid medial and mid lateral incisions (Figure 1 and Figure 2).With medial approach, through the subcutaneous tissue the ulnar nerve isolated and care taken to preserve brachial vessels as they can change their course with altered anatomy, and the medial condyle of humerus reached. Throughout the procedure, utmost care taken to stay subperiosteally and sticking on the supracondylar ridge both anterior and posterior elbow, to avoid any neurovascular injury. We used gauze piece, made into peanuts, to elevate the periosteum anteriorly and posteriorly. With lateral approach and similar technique, global soft tissue release was done all around 360°in continuity. The original technique of excision arthroplasty involved subperiosteal transverse resection at condylar level and some form of interpositional material was used. In our technique, the bony ankylosed elbow was osteotomised in an inverted 'V' shape(Figure 3 and Figure 4 and Figure 5) at the widest part of the bone,and no interpositional material was used as they have consistently given poor results and have carried risk of infection, donor site morbidity and foreign material reactions[12]. After completion of osteotomy, the anterior bony edges were smoothened and beveled more to increase the flexion of the elbow. Care should be taken to avoid overzealous excision of bone, to prevent floppy elbow. The bone edges smoothened using a burr with care to maintain optimum tension of soft tissue. Thus, medial and lateral stability was maintained because of intact sleeve of soft tissue released globally around the elbow and the V shaped osteotomy. Maximum flexion and extension obtained by trimming of the bone edges. All the patients achieved complete arc of flexion and extension intraoperatively. Arc of rotation also was checked. Two patients underwent excision of radial head who had limited arc of rotation after the osteotomy. Though many articles suggest many adjunctive methods of reconstruction during the procedure and stabilization post operatively but the unique osteotomy allows the joint surfaces to act as a hinge giving good stability and range of motion and we always believed in patients active involvement in active flexion and extension excercises which boosted patients energy levels which resulted in early satisfactory range of motion and comfort. Wound closed in layers with a suction drain after obtaining hemostasis, after releasing the tourniquet. First generation cephalosporin (cefazolin) was given as prophylactic antibiotic, one dose preoperatively and two doses postoperatively, at 8 hourly intervals, for all the patients. Elbow immobilized in 90°with crammer wire splint. Postoperatively, on day 1, controlled range of movement started from 90°to full flexion by manually distracting the joint by physiotherapist. Distraction flexion and extension excersies where patient is taught active flexion and extension with mild traction and counter traction with the help of physiotherapist. Distraction flexion method was taught to the patient and encouraged to do on their own. Post operative continuous regional analgesia in the intra scalene region with the catheter for 3 to 5 days depending on pain tolerability of the patient, patients were compliant with our exercise program. Sutures were removed on 12th day. Splint was continued till 3rd week. Progressive extension of the elbow started after 3 weeks. Until 6 weeks the splint was used as a rest splint.

**Figure 6:**
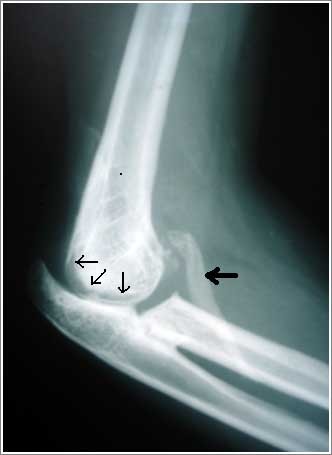
**Postoperative lateral radiograph of 28 year female showing the well formed elbow joint(marked by thin black arrows) with unexcised static HO anteriorly (shown by thick black arrow) at 8 months follow up**.

**Figure 7:**
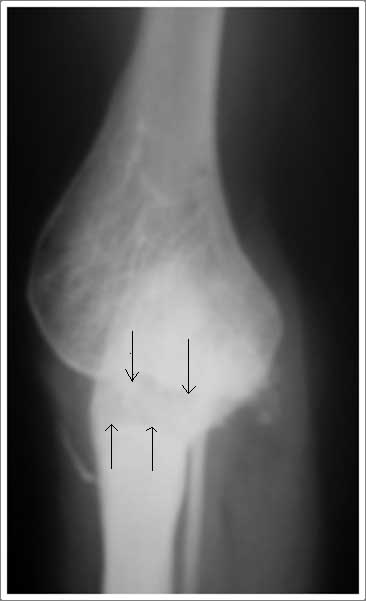
**Postoperative antero-posterior radiograph of 28 year female showing the rectangle radiolucent joint line marked by thin black arrows at 80 months follow up**.

The range of motion was measured using hand held goniometer. The patients were clinically evaluated using Mayo's elbow performance score[13,14]. It consists of four components: pain (maximum score, 45 points), motion (maximum score, 20 points), stability (maximum score, 10 points), and daily functional activities (maximum score, 25 points). A score of 90-100 is considered as excellent result; 75-89,as good result; 60-74, as fair result; less than 60,as poor result. The data were collected from the hospital medical records. Radiograph evaluation was done with two views; antero-posterior and lateral views(Figure 6 and Figure 7). The Mayo elbow performance score was calculated preoperatively and at final follow up, and radiographs taken preoperatively and at final follow up were assessed. The mean duration of follow up was 65 months (45 months -80 months).

## Results

**Figure 8:**
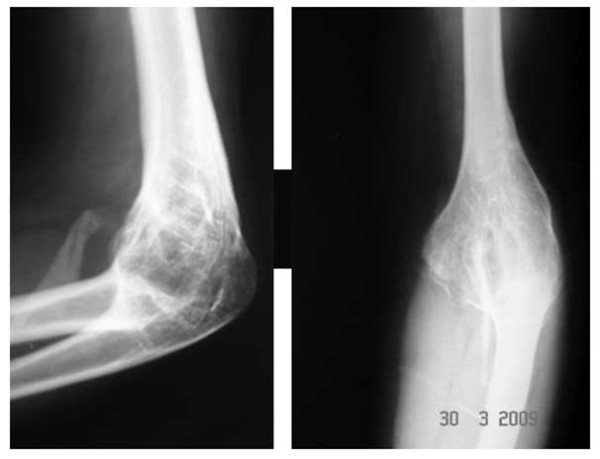
**Preoperative lateral and antero-posterior radiograph of 28 year Female demonstrating bony ankylosed elbow with matured HO anteriorly**.

**Figure 9:**
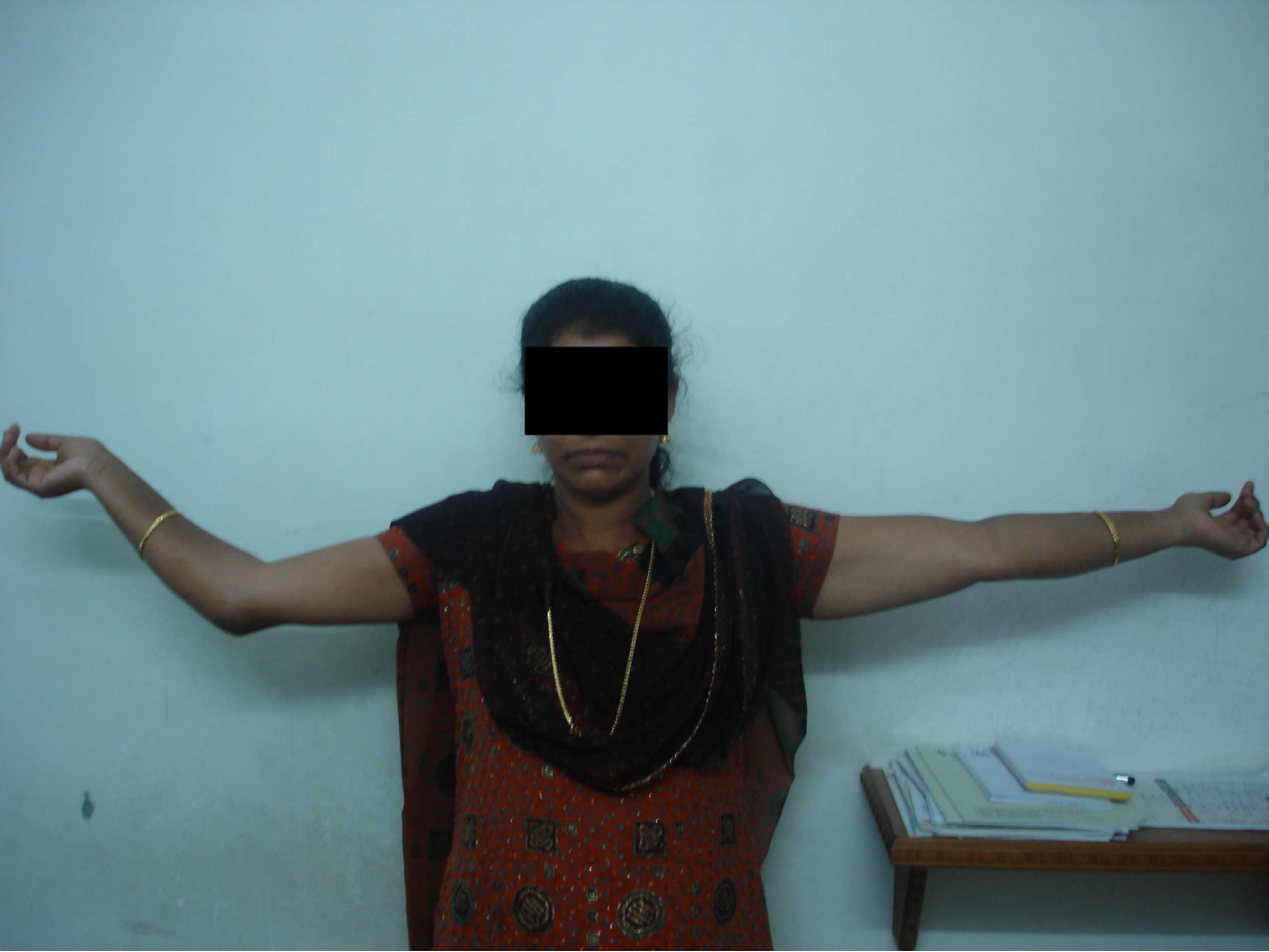
**Clinical photograph of 28 year female showing the final extension at 80 months follow up**.

**Figure 10:**
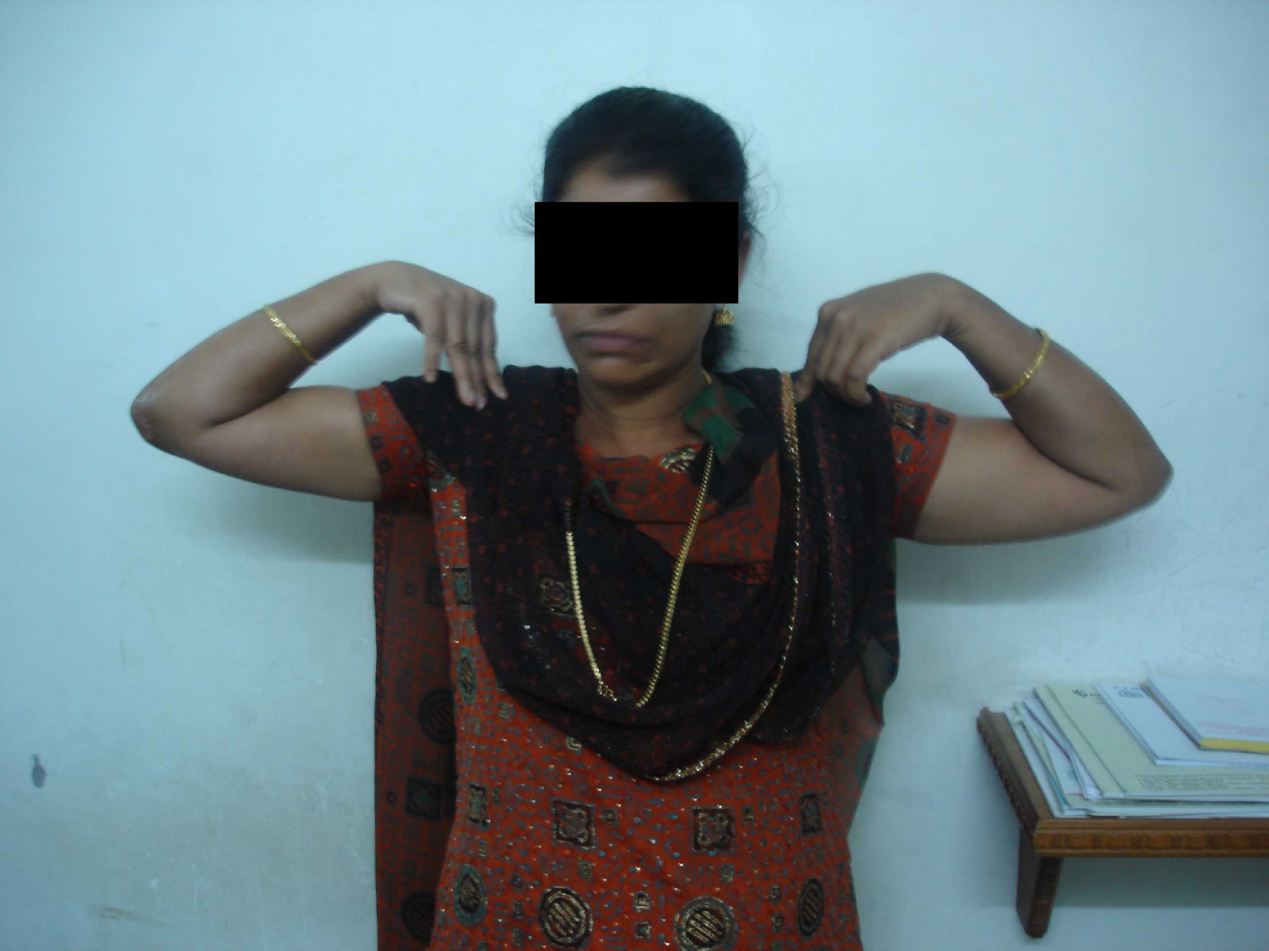
**Clinical photograph of 28 year female showing the final flexion at 80 months follow up**.

The mean preoperative position of elbow was 64 degrees (30°to 100^0^) with fixed elbow in all patients(Figure 8). The mean post operative range of motion at final follow up was 27°of extension (20^0^-50°), 116°of flexion (110^0^-130°), and the mean arc of motion was 88°(80^0^-100°) (table 1)(Figure 9 and Figure 10). The arc of rotation increased to an average of 81°(supination of 38^0^, pronation 42^0^) from the pre operative value of 47°(supination of 23^0^, pronation 24^0^), with three elbows with fixed rotation gaining mean arc of 41°(supination 16^0^, pronation 26°).

The mean Mayo's elbow performance score preoperatively was 48 (35-70). The MEPS at final follow up was 80 (60-95) (table 2). The improvement in arc of motion and MEPS score is statistically significant (p < 0.001). The heterotrophic ossification (HO) adjacent to anterior neurovascular structure was left undisturbed, as the patients had intraoperative gain of full functional range after the osteotomy, to avoid inadvertent injury to neurovascular structures. It remained static in the final radiograph with no new HO formation. No patient reported clinical instability though there was some subtle laxity on medial and lateral side on clinical examination, in comparison with the other normal elbow, that did not affect the functional activities of the patient and the MEPS. All patients were asymptomatic except one patient, who had pain mainly on heavy activities. All the patients were satisfied cosmetically and functionally. One patient had ulnar nerve neuropraxia and another patient developed median nerve neuropraxia, and both recovered completely in six weeks.

**Table 2:** pre operative and post operative arc of rotation and MEPS score.

No	supination	pronation	supination at final follow up	pronation at final follow up	arc of rotation at final follow up	pre op MEPS	MEPS at final follow up
1	0	0	20	30	50	50	85

2	30	40	40	40	80	35	80

3	30	30	70	40	110	50	85

4	50	30	50	50	100	70	95

5	30	-30	30	10	40	55	75

6	40	20	40	40	80	45	80

7	10	20	40	50	90	35	60

8	-40	40	0	40	40	45	80

9	60	70	60	80	140	50	85

## Discussion

Bony ankylosis of elbow is caused by plethora of causes, and remains a difficult problem to treat. The bony ankylosis of the elbow compromises functional ability of the arm, and puts greater demand on shoulder, spine, and wrist, as in the case of arthrodesis. The compensatory movement is more on spine and wrist, rather than the shoulder[15]. The primary indication for treating these patients is functional disability, though some patients present with upper limb pain. The challenge lies in treating such patients as the options are limited and are associated with complications. The options are resection arthroplasty, and total elbow replacement.

• Various reports are available in the literature regarding the use of excision arthroplasty for the treatment of ankylosis of the elbow, mainly fibrous ankylosis. However, the reports are limited for the treatment of bony ankylosis of the elbow, exclusively. Different kind of materials are interpositioned, such as fascia lata[16], muscle and capsule[17], fat[18], dermis[19], acrylic[20], nylon, homografts[21], and allografts[22]. The gel foam has been used commonly in two series of excision arthroplasty[10,23], and it has been mentioned that the operated limb becomes heavier for the patient as it adsorbs blood and body fluids. This makes uncomfortable for the patients and hence great effort is needed from the patient part to cooperate with post operative mobilization. We have not used any interpostional material in our patients. The gain in arc of motion after excision arthroplasty[10,11,24,25], and total elbow replacement [6,26] for bony ankylosis, reported in literature have all been significant, and increased the functional activity of the patient. In our series, the mean post operative range of motion at final follow up was 27°of extension (20^0^-50^0^), 116°of flexion (110^0^-130^0^), and the mean arc of motion was 88°(80^0^-100^0^), which is comparable to other series.

The pain was not significant feature in these patients compared to the functional disability. However, a few patients had mild pain or pain on exertion and they all were able to manage without affecting functional activity of life[10,11]. In our series, all but one patient had pain, mainly on heavy activities.

All the patients had perceptible lateral instability in most of the series[10,11] reported on literature on excision arthroplasty limiting functional activities. In our series, though patients had subtle medial and lateral instability on clinical examination, no patient reported functional instability as our surgical technique allows us to maintain global soft tissue sleeve, and inverted bony V cut provided additional bony stability. So, our technique provided better results in terms of stability, and better MEPS score. However, the surgical technique requires meticulous dissection as the anatomy could have been distorted due to longstanding bony ankylosis. We had one case of ulnar nerve neuropraxia, and another patient had median nerve neuropraxia. The ulnar nerve has been reported to be involved commonly in the literature[19,21,24,25], where as the median nerve involvement is rare. We possibly had one case of median nerve involvement due to inadvertent force while retracting during surgery.

Despite high possible risk of recurrence of HO, the benefit of prophylaxis remains subject of debate in case of elbow. In reported literature on excision arthroplasty, no method of prophylaxis was used[10,11,23-25]. David Ring et al[25] had two cases of recurrence of complete ankylosis out of 20 cases in their study, which was excised again and prophylactic radiotherapy was given. Due to limitation of resources, our patients were not given radiotherapy. In our patients Indomethacin 25 mg thrice a day, along with proton pump inhibitor, pantaprazole 40 mg once a day, was given for 4 weeks. No patients had recurrence of heterotopic ossification.

Several series available on Total elbow arthroplasty have included few cases of bony ankylosis [6,26-31]. B.F.Morey et al [6] in their series have reported their results on ten patients at final follow up with MEPS score of 74 points (50-95), with excellent in one, good in six, fair in three, poor in one. The complication rate in their series were notable, such as intra op fracture in two patients, malpositioning of the components, perioperative complication like soft tissue breakdown in two, and infection in one. Thus patients in their series required further surgeries for skin necrosis, infection, and implant loosening. In comparison, our series had comparable functional results in terms of MEPS score, with less complication rate and no revision surgery.

The limitation in our study is that it is retrospective one with less number of cohorts, and medium term follow up. The expectation of our patients was mainly for resumption of eating, drinking, and personal hygienic activities and hence our patients were satisfied functionally. Thus, we conclude from our results that Modified inverted "V" osteotomy excision arthroplasty is a viable option, especially in developing countries due to its limited resources.

No benefits in any form have been received or will be received from a commercial party related directly or indirectly to the subject of this article.

## Competing interests

The authors declare that they have no competing interests.

## Authors' contributions

CR was the person whoperformed the surgeries and a guide, SB RP were involved in drafting, PC SA and SR were involved in the documentation drafting and tabulating the data. All the authors have read and approved the final manuscript.
